# Content Swapping: A New Image Synthesis for Construction Sign Detection in Autonomous Vehicles

**DOI:** 10.3390/s22093494

**Published:** 2022-05-04

**Authors:** Hongje Seong, Seunghyun Baik, Youngjo Lee, Suhyeon Lee, Euntai Kim

**Affiliations:** School of Electrical and Electronic Engineering, Yonsei University, Seoul 03722, Korea; hjseong@yonsei.ac.kr (H.S.); shbaik104@yonsei.ac.kr (S.B.); lzozo95@yonsei.ac.kr (Y.L.); hyeon93@yonsei.ac.kr (S.L.)

**Keywords:** construction sign detection, image synthesis, cut-and-paste, perspective transformation

## Abstract

Construction signs alert drivers to the dangers of abnormally blocked roads. In the case of autonomous vehicles, construction signs should be detected automatically to prevent accidents. One might think that we can accomplish the goal easily using the popular deep-learning-based detectors, but it is not the case. To train the deep learning detectors to detect construction signs, we need a large amount of training images which contain construction signs. However, collecting training images including construction signs is very difficult in the real world because construction events do not occur frequently. To make matters worse, the construction signs might have dozens of different construction signs (i.e., contents). To address this problem, we propose a new method named content swapping. Our content swapping divides a construction sign into two parts: the board and the frame. Content swapping generates numerous synthetic construction signs by combining the board images (i.e., contents) taken from the in-domain images and the frames (i.e., geometric shapes) taken from the out-domain images. The generated synthetic construction signs are then added to the background road images via the cut-and-paste mechanism, increasing the number of training images. Furthermore, three fine-tuning methods regarding the region, size, and color of the construction signs are developed to make the generated training images look more realistic. To validate our approach, we applied our method to real-world images captured in South Korea. Finally, we achieve an average precision (AP_50_) score of 84.98%, which surpasses that of the off-the-shelf method by 9.15%. Full experimental results are available online as a supplemental video. The images used in the experiments are also released as a new dataset CSS138 for the benefit of the autonomous driving community.

## 1. Introduction

The misdetection of a construction sign may lead to accidents by unexpectedly entering blocked roads. Therefore, the reliable detection of construction signs is quite important in realizing autonomous driving. With the recent progress in object detection based on deep learning [1,2,3,4,5,6], one might think that we can accomplish the reliable detection of construction signs easily, but it is not true. To train the deep learning detector, we need large-scale training images including construction signs for robust and high-quality results. Unfortunately, construction signs appear infrequently on roads. Thus, collecting large amounts of training data for construction sign detection is required, but it is time-consuming and expensive. To address this problem, we propose a new method for learning to detect construction signs on roads. The main idea of the proposed method is to synthesize training images using a small number of construction sign images. To synthesize training images, we follow the cut-and-paste mechanism [7,8,9], which cuts an instance from the source image (i.e., construction sign region in an image) and pastes it into a background image (i.e., road image). The cut-and-paste method enables a model to avoid overfitting on a small number of backgrounds in source images, but it cannot generalize limited instances.

**Figure 1:**
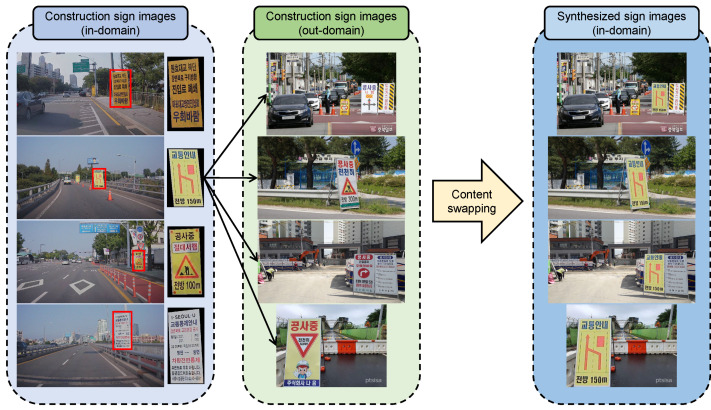
Content swapping. With $N_{I}$ in-domain and $N_{O}$ out-domain construction sign images, we synthesize $N_{I}N_{O}$ in-domain construction sign images via perspective transformation. The synthesized sign images are used as source images for cut-and-paste.

A construction sign can be divided into two parts: the board and frame. The content of the sign is contained in a rectangular board, and the board is supported by a frame. The frame can be shared for any sign. Using this characteristic, we effectively generate new construction sign images by swapping the contents in the rectangular board between two different construction sign images, as shown in Figure 1. Our content swapping synthesizes numerous synthetic construction signs by combining the board images (i.e., contents) taken from the in-domain images and the frames (i.e., geometric shapes) taken from the out-domain images. This approach allows us to obtain new $N_{I}N_{O}$ images from $N_{I}$ in-domain construction sign images and $N_{O}$ out-domain construction sign images. Although in-domain sign images need to be collected using the same camera setting as in the test set, out-domain images can be collected from the Internet. Therefore, we can synthesize a large-scale training dataset with only a small number of in-domain sign images and train a detector on them.

We also develop three fine-tuning methods to improve the quality of synthetic training images. The three methods deal with the (1) pasted region, (2) instance size, and (3) color difference of the synthesized images, respectively. The first method guides us to paste the synthetic construction sign image on the drivable region. Because the construction sign cannot be placed on the sky, car, or other objects, it should be placed only on the drivable region for realistic purposes. The second method helps us to select the size of the instance based on the location where the sign is to be pasted. If we assume that the construction sign is always pasted on the road and the road is flat, then we can automatically predict the size of the instance in the image. The prediction not only avoids making construction images either too large or too small but also resizes the images to match nearby objects, thereby improving global consistency. Finally, we blend the synthesized construction signs with the training image to reduce the gap between the source and background images. The blending also reduces the domain gap between in-domain and out-domain construction sign images in content swapping. To our best knowledge, no other research has been conducted to detect the construction signs. To validate the effectiveness of the proposed methods, we collect the CSS138 (Construction Signs in Seoul with 138 images) dataset for training and testing construction sign detection. All the images are captured in Seoul, Korea. The CSS138 dataset can be downloaded at https://github.com/Hongje/content-swapping, (accessed on 5 April 2022). In the experiment, we synthesize a large-scale training dataset with only 12 in-domain sign images and achieve a robust and accurate result with an AP_50_ score of 84.98% for CSS138. Our result surpasses off-the-shelf cut-and-paste by 9.15% in the AP_50_ score. Full experimental results are available online: https://youtu.be/us_qso6C5pw, (accessed on 5 April 2022).

The main contributions are summarized as follows:

- This is the first paper which deals with the *construction* sign detection.
- We propose a new image synthesis method, content swapping, to avoid overfitting on limited instances in source images.
- We further present three fine-tunning methods for creating realistic construction images on roads.
- To demonstrate the efficacy of the proposed method, we construct a new dataset, CSS138, for construction sign detection.
- Finally, we achieve an AP_50_ score of 84.98%, creating a gap of 9.15% from the naive cut-and-paste method.

The remainder of this paper is organized as follows. Previous works related to this study are discussed in Section 2. The proposed method for synthesizing construction images is described in Section 3. The experimental results for CSS138 and the analysis are presented in Section 4. Finally, the conclusions are presented in Section 5.

## 2. Related Work

### 2.1. Sign Detection

Early methods designed models for detecting signs heuristically. Specifically, Prince et al. [10] design a sign detection algorithm based on a geometrical analysis of the edges and groups of the sign image features. Escalera et al. [11] segment images using color thresholding and then analyze the shape to detect signs. Fang et al. [12] formulate three types of shapes—circular, triangular, and octagonal—to extract the color features of the signs. Shadeed et al. [13] convert the RGB color space to HSV and YUV color spaces and then defined a heuristic algorithm. Loy et al. [14] exploit the symmetric nature and the pattern of the edge of the triangular, square, and octagonal shapes to predict the shape of the sign image. Bahlmann et al. [15] propose a joint color and shape information modeling approach using a set of Haar wavelet features.

Recently, state-of-the-art approaches have used convolutional neural network (CNN)-based supervised models. Shao et al. [16] train CNNs with simplified Gabor filters. Cao et al. [17] use shallow CNNs to classify the traffic signs. Zhang et al. [18] propose a new cascaded R-CNN architecture that includes multiscale attention and imbalanced samples. Liu et al. [19] propose TSingNet, which is based on feature pyramid networks and includes several attention-based modules. Ahmed et al. [20] propose a new DNN-based framework that is robust in detecting traffic signs, even under challenging weather conditions. Zeng et al. [21] propose an improved YOLOv3 architecture for real-time traffic-sign detection. All previous methods considered only traffic or road signs.

The basic difference between general sign detection and construction sign detection is how much training samples are provided. Differently from the large amount of training images in general sign detection, only dozens of training images are given in construction sign detection. Furthermore, collecting the training images for construction signs is much more difficult. The key idea of our method is how to augment the training images and train a detector on them effectively. The purpose to detect and recognize construction signs is to alert the unplanned situations made by road construction. Understandably, commercial autonomous vehicles can handle not only the planned situations but also the unplanned situations. The typical example of the unplanned situation might be the road construction. In this case, the autonomous vehicle may not have to obey the traffic law. For example, our vehicle may have to cross the road following policeman’s hand signal, ignoring the traffic sign. The goal of our paper is to handle that kind of unplanned abnormal situation.

Our construction sign detection can also be considered as a special kind of class imbalance problem. We are dealing with only a single class (i.e., construction sign) and the instances of the class are highly imbalanced with the background instances such as buildings, roads, or pedestrians. The key idea of the paper is to tackle the serious imbalance problem by augmenting the training samples.

### 2.2. Image Synthesis for Network Training

Several studies [9,22] have synthesized training images with a focus on realism. Furthermore, task-specific image synthesis has also been extensively studied. Dwibedi et al. [7] propose a simple yet effective training image synthesis method that uses cut-and-paste for object detection. Lee et al. [8] propose content transfer, which transfers tail-class content from source to target to address the class imbalance problem in unsupervised domain-adaptive semantic segmentation. Leon et al. [9] synthesize training images by rendering that does not require real-world images. In this paper, we propose methods for synthesizing construction sign images for sign detection. The key idea of our image synthesis is that the contents of the board are taken from in-domain images, whereas the frame is taken from the out-domain (and in-domain) images. Since the frames includes only the geometrical shape of the sign board, they can be collected from any images (out-domain images) without affecting the detection performance. However, since the board images have their own style, the construction sign images taken only from the in-domain images are used to facilitate the synthesis onto the background road images.

## 3. Method

### 3.1. Overview

**Figure 2:**
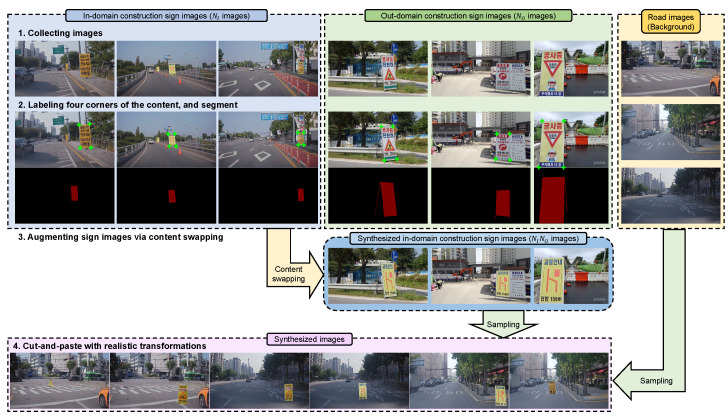
An overview of training data synthesis. The entire process was divided into four steps. First, we collected three types of images: in-domain construction sign images, out-domain construction sign images, and road images. Then, we labeled four corners of the contents and segmented the construction sign images. The labels were then used for content swapping. Finally, a pair of a construction image and a road image was randomly sampled and synthesized via the cut-and-paste mechanism with proposed realistic transformations. The synthesized images are used for training networks.

An overview of the proposed method for synthesizing training data is shown in Figure 2. The entire process of synthesizing the training images comprised four main steps. In the first step, we prepared images by collecting construction sign images and road images. As acquiring construction sign images is difficult, we could only prepare a limited number of sign images. Therefore, we collected additional out-domain construction sign images from the Internet. In the second step, the four corners of the content and segmentation mask were labeled in the construction sign images. In the third step, content swapping was performed using these labels. Finally, the training images were generated via the cut-and-paste mechanism using the proposed realistic transformations.

For a clearer explanation, we provide a pseudo-code of the proposed method in Algorithm 1. Each step in Algorithm 1 matches Figure 2. In the following subsections, we describe the details of each step.
**Algorithm 1** Pseudo-code of the proposed method.**Step1: Collecting construction sign and road images**1: In-domain construction sign image: $I_{In}$2: Out-domain construction sign image: $I_{Out}$3: Road image: $I_{Road}$**Step2: Labeling bounding box, segment, and four corners of the board**4: Bounding box labels: $Bbox_{In}$, $Bbox_{Out}$5: Segment labels: $M_{In}$, $M_{Out}$6: Four corners of the board: $\left(\left[\begin{array}{cc} x_{I}^{1} & y_{I}^{1} \end{array}\right]\left[\begin{array}{cc} x_{I}^{2} & y_{I}^{2} \end{array}\right]\left[\begin{array}{cc} x_{I}^{3} & y_{I}^{3} \end{array}\right]\left[\begin{array}{cc} x_{I}^{4} & y_{I}^{4} \end{array}\right]\right)$, $\left(\left[\begin{array}{cc} x_{O}^{1} & y_{O}^{1} \end{array}\right]\left[\begin{array}{cc} x_{O}^{2} & y_{O}^{2} \end{array}\right]\left[\begin{array}{cc} x_{O}^{3} & y_{O}^{3} \end{array}\right]\left[\begin{array}{cc} x_{O}^{4} & y_{O}^{4} \end{array}\right]\right)$**Step3: Content swapping**7: Randomly select content image (source): S∈In8: Randomly select frame image (target): $T\in \left[\begin{array}{cc} In & Out \end{array}\right]$9: Set content region mask of target image using four corners label: $C_{T}$10: Compute transformation matrix T:      ▹ Section 3.4$T={\left[\begin{array}{cccccccc} x_{S}^{1} & y_{S}^{1} & 1 & 0 & 0 & 0 & -x_{S}^{1}x_{T}^{1} & -y_{S}^{1}x_{T}^{1}\\ 0 & 0 & 0 & x_{S}^{1} & y_{S}^{1} & 1 & -x_{S}^{1}y_{T}^{1} & -y_{S}^{1}y_{T}^{1}\\ x_{S}^{2} & y_{S}^{2} & 1 & 0 & 0 & 0 & -x_{S}^{2}x_{T}^{2} & -y_{S}^{2}x_{T}^{2}\\ 0 & 0 & 0 & x_{S}^{2} & y_{S}^{2} & 1 & -x_{S}^{2}y_{T}^{2} & -y_{S}^{2}y_{T}^{2}\\ x_{S}^{3} & y_{S}^{3} & 1 & 0 & 0 & 0 & -x_{S}^{3}x_{T}^{3} & -y_{S}^{3}x_{T}^{3}\\ 0 & 0 & 0 & x_{S}^{3} & y_{S}^{3} & 1 & -x_{S}^{3}y_{T}^{3} & -y_{S}^{3}y_{T}^{3}\\ x_{S}^{4} & y_{S}^{4} & 1 & 0 & 0 & 0 & -x_{S}^{4}x_{T}^{4} & -y_{S}^{4}x_{T}^{4}\\ 0 & 0 & 0 & x_{S}^{4} & y_{S}^{4} & 1 & -x_{S}^{4}y_{T}^{4} & -y_{S}^{4}y_{T}^{4} \end{array}\right]}^{-1}\left[\begin{array}{c} x_{T}^{1}\\ y_{T}^{1}\\ x_{T}^{2}\\ y_{T}^{2}\\ x_{T}^{3}\\ y_{T}^{3}\\ x_{T}^{4}\\ y_{T}^{4} \end{array}\right]$11: Swap content: $I_{T}^{CS}=T\left(I_{S}\right)⊙C_{T}+I_{T}⊙\left(1-C_{T}\right)$**Step4: Cut-and-paste with realistic transformations**12: Randomly select road image (background): B∈Road13: Compute pasteable region: $P_{B}$    ▹ Section 3.5.114: Randomly select bottom point of the sign: $p_{1}=\left[\begin{array}{cc} p_{1}^{x} & p_{1}^{y} \end{array}\right]\in P_{B}$15: Compute top point of the sign:    ▹ Section 3.5.2p2=p2xp2y=p1xtan−1tanα·p1y+β1−h−βtan−1tanα·p1y+β1−h−βαα16: Cut sign image $I_{T}^{CS}$ and paste to road image $I_{B}$: $I_{T}^{CP}=\mathrm{Cut}-\mathrm{and}-\mathrm{Paste}\left(I_{T}^{CS},M_{T},I_{B},p_{1},p_{2}\right)$17: Transform segment label to $p_{1}$ and $p_{2}$: $M_{T}^{CP}=\mathrm{Transform}\_\mathrm{mask}\left(M_{T},p_{1},p_{2}\right)$18: Transform bounding box label to $p_{1}$ and $p_{2}$: $Bbox_{T}^{CP}=\mathrm{Transform}\_\mathrm{box}\left(Bbox_{T},p_{1},p_{2}\right)$19: Reduce color difference: $I_{T}^{GP}=\mathrm{GP}-\mathrm{GAN}\left(I_{T}^{CS},M_{T}^{CS}\right)$    ▹ Section 3.5.3**Output:** Synthesized training image: $I_{T}^{GP}$;      Synthesized training label: $Bbox_{T}^{CP}$

### 3.2. Collecting Images

**Figure 3:**
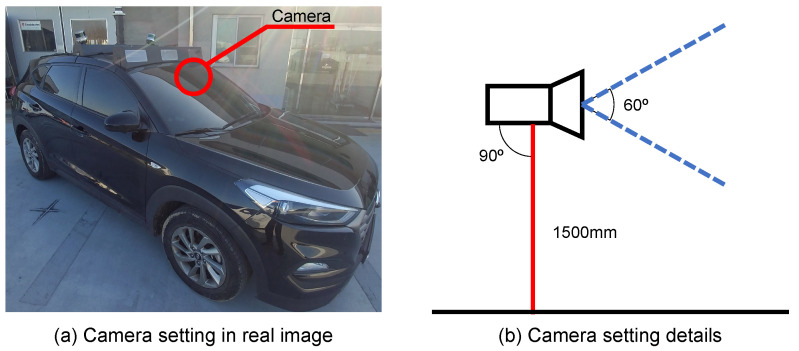
The camera setting in the data-collecting platform.

To collect construction sign and road images, we used the FHD390C-USB(D) (Autonomous A2Z, Gyeongsan, South Korea) camera model. This model captures full HD images (1080p) in 30 frames per second. It has a field of view of 60 degrees. We built a data-collecting platform using this camera model, as shown in Figure 3. The camera was installed at a height of 1500mm from the ground and was positioned in front of a platform so that we could collect front-view images of the roads. In total, we collected 138 construction sign images, of which 12 were used for training and the remaining 126 were used for testing. The collected construction sign images were used as in-domain images. In addition, we collected 992 road images that did not contain any construction signs. All the images were captured in Seoul, Korea. Twelve images were used to collect the contents of the construction signs. We also collected an additional 24 construction sign images from the Internet. They were out-domain construction sign images, and they were used to capture the frame of the construction sign boards.

**Figure 4:**
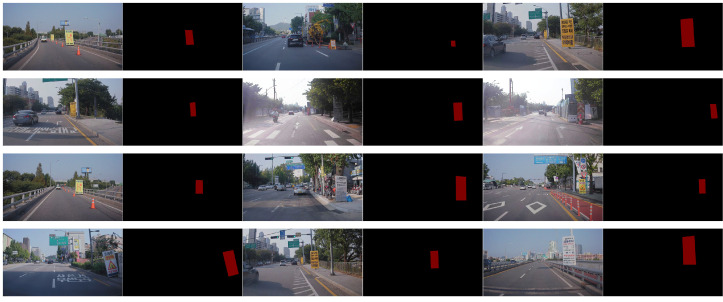
Collected in-domain construction signs.

We collected 12 construction signs using our platform. Thus, we had 12 kinds of construction signs (12 in-domain images) for the board region. We also gathered 24 construction sign images from the Internet, making 36 kinds of construction signs (12 in-domain + 24 out-domain images) for the frame region. The collected 12 in-domain construction signs are shown in Figure 4.

### 3.3. Labeling

We annotate three types of labels in the construction sign images. First, we annotate the bounding box for all the collected construction sign images. Bounding box annotations are needed to compute the loss during training and evaluate the detection quality during testing. Second, we annotate the four corners of the board in the training set of construction sign images. Corner annotation is required to calculate the transformation matrix between two construction sign images. Third, we annotate the per-pixel label of the construction sign. Pixel-level annotations are used for both content swapping (detailed in Section 3.4) and cut-and-paste (detailed in Section 3.5).

### 3.4. Content Swapping

To overcome the lack of the training image, we synthesize training images using a cut-and-paste [7,8,9] mechanism, as shown in Figure 1. The cut-and-paste effectively helps to prevent the networks from overfitting on the limited backgrounds of the training images. However, the cut-and-paste method cannot augment the content of the training images. This means that only background images can be diversified, and the contents of the construction signs are still limited. We address this problem using content swapping.

**Figure 5:**
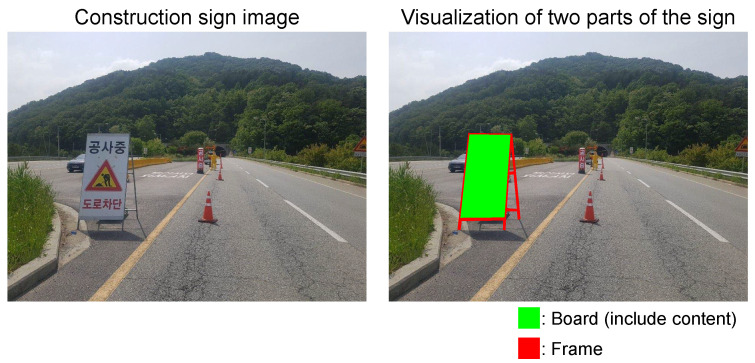
Visualization of the two parts of the construction sign. In the first column, we show a construction sign image. In the second column, we denote the board and frame regions with green and red, respectively.

The construction sign can be divided into two parts: a rectangular board and frame, as shown in Figure 5. Therefore, we can reuse the frame for other constructions by replacing only the board. To replace the board in the target sign image with the source sign image, we need to formulate the transformation function between the source image and the target image. Thankfully, because the shape of the board is rectangular, replacing the content is possible with four pairs of corner points on the board using perspective transformation, as follows:

(1) $$\left[\begin{array}{c} wx_{T}\\ wy_{T}\\ w \end{array}\right]=T\left[\begin{array}{c} x_{S}\\ y_{S}\\ 1 \end{array}\right]=\left[\begin{array}{ccc} p_{11} & p_{12} & p_{13}\\ p_{21} & p_{22} & p_{23}\\ p_{31} & p_{32} & 1 \end{array}\right]\left[\begin{array}{c} x_{S}\\ y_{S}\\ 1 \end{array}\right]$$

where ${\left[\begin{array}{cc} x_{S} & y_{S} \end{array}\right]}^{T}$ and ${\left[\begin{array}{cc} x_{T} & y_{T} \end{array}\right]}^{T}$ are the source and target points of the construction sign images, respectively, and $T=\left[\begin{array}{ccc} p_{11} & p_{12} & p_{13}\\ p_{21} & p_{22} & p_{23}\\ p_{31} & p_{32} & 1 \end{array}\right]$ is the perspective transformation matrix with 8 parameters. Here, the eight parameters in the perspective transformation matrix T are unknown. Then, we can unfold the equations as:

(2) $$x_{T}=\left(p_{11}x_{S}+p_{12}y_{S}+p_{13}\right)/w,$$

(3) $$y_{T}=\left(p_{21}x_{S}+p_{22}y_{S}+p_{23}\right)/w,$$

(4) $$w=p_{31}x_{S}+p_{32}y_{S}+1.$$

By substituting Equation (4) into Equations (2) and (3), we can join a parameter *w* into $x_{T}$ and $y_{T}$ as:

(5) $$x_{T}=\frac{p_{11}x_{S}+p_{12}y_{S}+p_{13}}{p_{31}x_{S}+p_{32}y_{S}+1},$$

(6) $$y_{T}=\frac{p_{21}x_{S}+p_{22}y_{S}+p_{23}}{p_{31}x_{S}+p_{32}y_{S}+1}.$$

To easily formulate each unknown parameter in T into a matrix form, we can rearrange Equations (5) and (6) into:

(7) $$x_{T}=p_{11}x_{S}+p_{12}y_{S}+p_{13}-p_{31}x_{S}x_{T}-p_{32}y_{S}x_{T},$$

(8) $$y_{T}=p_{21}x_{S}+p_{22}y_{S}+p_{23}-p_{31}x_{S}y_{T}-p_{32}y_{S}y_{T},$$

respectively. Here, there are eight unknown parameters (i.e., $\left[\begin{array}{cccc} p_{11} & p_{12} & \ldots & p_{32} \end{array}\right]$). Therefore, to estimate the eight parameters’ values, we need eight different formulas. With Equations (7) and (8), we can make eight different formulas using four known pairs of corresponding points ($\left[\begin{array}{cc} x_{S}^{1} & y_{S}^{1} \end{array}\right]\ldots \left[\begin{array}{cc} x_{S}^{4} & y_{S}^{4} \end{array}\right]$ for source points and $\left[\begin{array}{cc} x_{T}^{1} & y_{T}^{1} \end{array}\right]\ldots \left[\begin{array}{cc} x_{T}^{4} & y_{T}^{4} \end{array}\right]$ for target points), and then we can write them into a matrix as follows:

(9) $$\left[\begin{array}{c} x_{T}^{1}\\ y_{T}^{1}\\ x_{T}^{2}\\ y_{T}^{2}\\ x_{T}^{3}\\ y_{T}^{3}\\ x_{T}^{4}\\ y_{T}^{4} \end{array}\right]=\left[\begin{array}{cccccccc} x_{S}^{1} & y_{S}^{1} & 1 & 0 & 0 & 0 & -x_{S}^{1}x_{T}^{1} & -y_{S}^{1}x_{T}^{1}\\ 0 & 0 & 0 & x_{S}^{1} & y_{S}^{1} & 1 & -x_{S}^{1}y_{T}^{1} & -y_{S}^{1}y_{T}^{1}\\ x_{S}^{2} & y_{S}^{2} & 1 & 0 & 0 & 0 & -x_{S}^{2}x_{T}^{2} & -y_{S}^{2}x_{T}^{2}\\ 0 & 0 & 0 & x_{S}^{2} & y_{S}^{2} & 1 & -x_{S}^{2}y_{T}^{2} & -y_{S}^{2}y_{T}^{2}\\ x_{S}^{3} & y_{S}^{3} & 1 & 0 & 0 & 0 & -x_{S}^{3}x_{T}^{3} & -y_{S}^{3}x_{T}^{3}\\ 0 & 0 & 0 & x_{S}^{3} & y_{S}^{3} & 1 & -x_{S}^{3}y_{T}^{3} & -y_{S}^{3}y_{T}^{3}\\ x_{S}^{4} & y_{S}^{4} & 1 & 0 & 0 & 0 & -x_{S}^{4}x_{T}^{4} & -y_{S}^{4}x_{T}^{4}\\ 0 & 0 & 0 & x_{S}^{4} & y_{S}^{4} & 1 & -x_{S}^{4}y_{T}^{4} & -y_{S}^{4}y_{T}^{4} \end{array}\right]\left[\begin{array}{c} p_{11}\\ p_{12}\\ p_{13}\\ p_{21}\\ p_{22}\\ p_{23}\\ p_{31}\\ p_{32} \end{array}\right].$$

The objective is to estimate eight unknown parameters in T. Therefore, we can finally obtain the transformation matrix T by computing the inverse of the 8 × 8 matrix in Equation (9) and performing matrix multiplication as follows:

(10) $${\left[\begin{array}{cccccccc} x_{S}^{1} & y_{S}^{1} & 1 & 0 & 0 & 0 & -x_{S}^{1}x_{T}^{1} & -y_{S}^{1}x_{T}^{1}\\ 0 & 0 & 0 & x_{S}^{1} & y_{S}^{1} & 1 & -x_{S}^{1}y_{T}^{1} & -y_{S}^{1}y_{T}^{1}\\ x_{S}^{2} & y_{S}^{2} & 1 & 0 & 0 & 0 & -x_{S}^{2}x_{T}^{2} & -y_{S}^{2}x_{T}^{2}\\ 0 & 0 & 0 & x_{S}^{2} & y_{S}^{2} & 1 & -x_{S}^{2}y_{T}^{2} & -y_{S}^{2}y_{T}^{2}\\ x_{S}^{3} & y_{S}^{3} & 1 & 0 & 0 & 0 & -x_{S}^{3}x_{T}^{3} & -y_{S}^{3}x_{T}^{3}\\ 0 & 0 & 0 & x_{S}^{3} & y_{S}^{3} & 1 & -x_{S}^{3}y_{T}^{3} & -y_{S}^{3}y_{T}^{3}\\ x_{S}^{4} & y_{S}^{4} & 1 & 0 & 0 & 0 & -x_{S}^{4}x_{T}^{4} & -y_{S}^{4}x_{T}^{4}\\ 0 & 0 & 0 & x_{S}^{4} & y_{S}^{4} & 1 & -x_{S}^{4}y_{T}^{4} & -y_{S}^{4}y_{T}^{4} \end{array}\right]}^{-1}\left[\begin{array}{c} x_{T}^{1}\\ y_{T}^{1}\\ x_{T}^{2}\\ y_{T}^{2}\\ x_{T}^{3}\\ y_{T}^{3}\\ x_{T}^{4}\\ y_{T}^{4} \end{array}\right]=\left[\begin{array}{c} p_{11}\\ p_{12}\\ p_{13}\\ p_{21}\\ p_{22}\\ p_{23}\\ p_{31}\\ p_{32} \end{array}\right].$$

Using the estimated transformation matrix T, we warped the board from the source image to the target image, which is called content swapping.

With content swapping, we can effectively augment in-domain construction sign images using out-domain construction sign images. Given the $N_{I}$ in-domain and $N_{O}$ out-domain construction sign images, we can synthesize in-domain images by content swapping from in-domain sign images to out-domain images, resulting in $N_{I}N_{O}$ pairs. Therefore, although we obtained only 12 in-domain construction sign images for training, 288 in-domain images can be obtained using 24 out-domain sign images. Furthermore, we use the frame region in the in-domain sign images for content swapping, which resulted in 432 construction sign images.

### 3.5. Cut-and-Paste with Realistic Transformations

We synthesize training images by cutting a construction sign image and then pasting it onto the background road images. Here, naively cutting and pasting would result in unrealistic synthetic images, which may lead to performance degradation. We address this problem by proposing three fine-tunning methods. They are developed from three perspectives: pasteable region, instance size, and color difference. Detailed explanations of each fine-tuning methods are provided below.

#### 3.5.1. Pasteable Region

The construction sign cannot fly and is never placed on a car. Therefore, we set the pasteable region as the road. To find road regions in the background image, we used two independent pre-trained networks: semantic segmentation and depth estimation. For the semantic segmentation network, we used DeepLab v3+, trained on Cityscapes https://www.cityscapes-dataset.com, (accessed on 5 April 2022). Because the road class is included in the Cityscapes dataset, the predicted score of the road is used directly. For the depth estimation network, we use the off-the-shelf depth prediction network, MiDaS [23]. The estimated depth is used to filter the noise by thresholding. Thus, the regions that are predicted as roads and with estimated depths lower than the predefined threshold are defined as pasteable regions.

#### 3.5.2. Instance Size

Close objects look large and far objects look small. This property is also preserved in the images. Using this property, we adjust the instance size of the construction sign according to the pasted position. In the image, we first randomly select a pixel within the pasteable region ($p_{1}=\left[\begin{array}{cc} p_{1}^{x} & p_{1}^{y} \end{array}\right]$). The selected pixel is the bottom point of the construction sign. In real-world coordinates, we compute the distance between the camera and the sign (*d*), under the assumption that the road is flat, as follows:

(11) $$d=H_{cam}\tan\theta_{1}$$

where $H_{cam}$ denotes the height of the camera from the road, and $\theta_{1}$ is the angle between the line from the camera to the road and the line from the camera to the bottom of the construction sign line. The angle $\theta_{1}$ is proportional to $p_{1}^{y}$:

(12) $$\theta_{1}=\alpha \cdot p_{1}^{y}+\beta$$

where α and β are constants. Given the computed distance *d*, we can calculate the angle $\theta_{2}$, which is the angle between the line from the camera to the road and the line from the camera to the top of the construction sign, as follows:

(13) $$\theta_{2}=\tan^{-1}\left(\frac{d}{H_{cam}-H_{sign}}\right)$$

where $H_{sign}$ denotes the height of the construction sign, and $H_{sign}<H_{cam}$. For simplification, we assume that all construction signs have the same height $H_{sign}$ and stand perpendicular to the road. Then, in the image coordinates, we compute the top point of the construction sign ($p_{2}=\left[\begin{array}{cc} p_{2}^{x} & p_{2}^{y} \end{array}\right]$) using the proportionality between $\theta_{2}$ and $p_{2}^{y}$ as follows:

(14) $$p_{2}^{x}=p_{1}^{x},$$

(15) p2y=(θ2−β)/α=tan−1dHcam−Hsign−βtan−1dHcam−Hsign−βαα=tan−1Hcamtanθ1Hcam−Hsign−βtan−1Hcamtanθ1Hcam−Hsign−βαα=tan−1Hcamtanα·p1y+βHcam−Hsign−βtan−1Hcamtanα·p1y+βHcam−Hsign−βαα.

**Figure 6:**
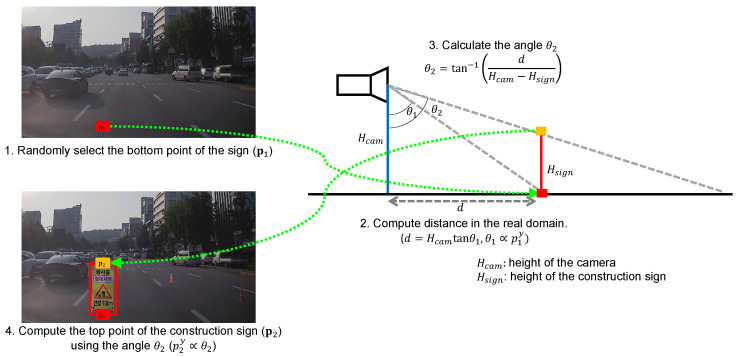
Step-by-step processes for computing the size of the construction sign. (1) We randomly select the bottom position of the construction sign in the image. The position is selected only within the pasteable region. (2) From the randomly selected point in the image, we estimate the angle $\theta_{1}$ and compute the distance between the camera and the sign in the real domain. (3) We estimate the angle $\theta_{2}$ by assuming that all construction signs have the same height $H_{sign}$ and stand perpendicular to the road. (4) Using the estimated angle $\theta_{2}$, we compute the point of the top of the construction sign in the image.

In Equation (15), we divide the denominator and numerator by $H_{cam}$ as:

(16) p2y=tan−1tanα·p1y+β1−Hsign/Hcam−βtan−1tanα·p1y+β1−Hsign/Hcam−βαα.=tan−1tanα·p1y+β1−h−βtan−1tanα·p1y+β1−h−βαα.

where *h* denotes the ratio of the height of the sign to the camera. Using Equations (14) and (16), the top point of the construction sign can be directly computed from the bottom point. We empirically set the parameters α, β, and *h* to π/3888, π/3, and 0.75, respectively. The overall process for computing the size of the construction signs is summarized in Figure 6.

#### 3.5.3. Color Difference

**Figure 7:**
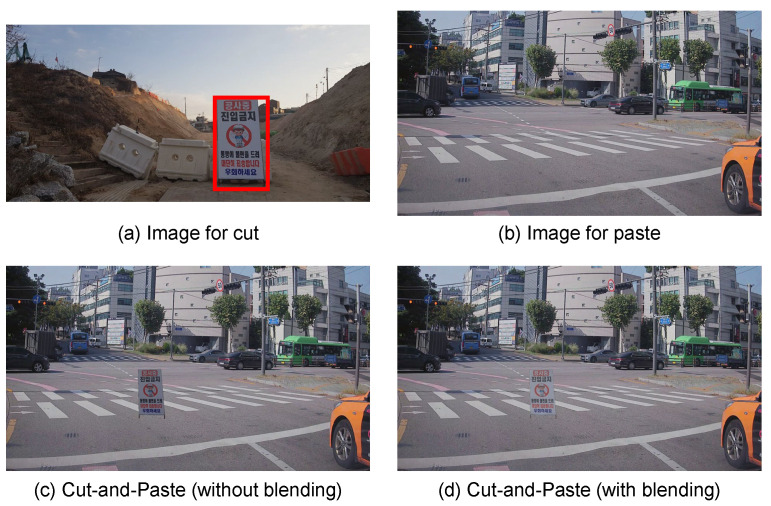
Effect of blending. Using the construction sign image in (**a**) and the road image in (**b**), we synthesize the training image via cut-and-paste. As shown in (**c**), however, the artifact seems prominent because of the color difference between the construction sign and the road. This problem is mitigated by blending, as shown in (**d**).

One of the main reasons for the artifacts in the synthesized images, which is made using cut-and-paste, is the color difference between the two images. As shown in Figure 7c, the color difference is caused by differences in illumination, weather, and environment. To match the color difference between the construction sign image and road image, we blend the synthesized image using an off-the-shelf model, GP-GAN [24]. By blending, we can reduce the artifacts of the synthesized image, as shown in Figure 7d.

## 4. Experiments

### 4.1. Implementation Details

We conduct some experiments using our collected construction sign detection dataset, CSS138. We use YOLOv3 [1] as a construction sign detector. Basically, we follow the training and inference details in the original YOLOv3 paper [1]. We use Darknet-53 [1] as a backbone network. Darknet-53 consists of 53 convolutional layers and 23 residual connections. Darknet-53 outputs three different sizes of features, which have 1/8, 1/16, and 1/32 resolutions with respect to the input image. To detect construction signs from encoder’s feature, a decoder is used. The decoder takes three outputs of Darknet-53, and outputs detection results at three different resolutions, i.e., 1/8, 1/16, and 1/32 resolutions with respect to the input image. Each output predicts five values: four for coordinates of the bounding box and one for objectness. Unlike vanilla YOLOv3, which predicts the class of the object, we do not predict the object class because we have only a single object class, construction sign, in this paper.

Additionally, we apply our method to YOLOv3-tiny to see the effectiveness of our method in other networks. YOLOv3-tiny uses Darknet-19 [25] as a backbone network. Darknet-19 has 19 convolutional layers without residual connections. YOLOv3 has 61.5M parameters, while YOLOv3-tiny has 8.7M parameters. These parameter numbers are comparable with state-of-the-art object detectors: Faster R-CNN [26] has 52.7M parameters, FPN [27] has 60.6M parameters, and RetinaNet [28] has 56.9M parameters. To train YOLOv3, we use an RGB image as an input.

A total of 9920 RGB images are synthesized for training using the CSS138 training set. The training set includes 992 road images and 36 construction sign images. Among 36 sign images, 12 images are in-domain and 24 are out-domain. We randomly crop 640 × 640 patches for the training. The learning rate is initially set to 1 × 10${}^{-2}$, and we decrease the learning rate to 1 × 10${}^{-3}$ using the cosine decay schedule. The network is trained in 375,000 iterations with a mini-batch size of 16. The entire training process takes approximately 60 h using a single NVIDIA Titan V GPU. During inference, we obtain multiple detection results at three different resolutions. To select accurate results and dismiss overlapped noisy results, we use non-maximal suppression with an IoU threshold of 0.45.

### 4.2. Quantitative Results

We validate our approach on the CSS138 validation set. The CSS138 validation set includes 126 images containing at least one construction sign. For quantitative evaluation, we measure the average precision with Intersection over Union (IoU) thresholds of 0.5, and we denote it as AP_50_. Following the recent object detection benchmark https://cocodataset.org/#detection-eval, (accessed on 5 April 2022), we additionally measure AP, which is calculated by computing 10 average precision values with IoU thresholds of {0.5, 0.55, …0.9, 0.95} and then averaging them. To demonstrate the superiority of our approach, we set a baseline that synthesizes 9920 training images by using a naive cut-and-paste method. From the baseline, we add the proposed methods in a step-by-step manner. The experimental results for the CSS138 validation set are listed in Table 1. As shown in Table 1, our method achieves AP and AP_50_ scores of 70.36% and 84.98%, respectively, whereas the baseline achieves scores of 60.53% and 75.84%, respectively. We surpass the baseline by >+9% for both the AP and AP_50_ scores. Table 1 shows the contributions of each step of the proposed method. Each step improves the performance by >+2% for both AP and AP_50_. This demonstrates that all our approaches are effective in synthesizing training images for construction sign detection.

In Table 2, we additionally validate the efficacy of our instance size adjustment method. As described in Section 3.5.2, we resize the instance by projecting it to real-world coordinates. We can compare it with Fixed, which uses the original scale of the construction sign image. We can further compare it with Random, which uses a randomly sampled value for scaling construction sign images and was used in cut-and-paste [7]. As shown in Table 2, we significantly surpass Fixed and Random by 5% and 2%, respectively, in terms of the AP_50_ score. The results demonstrate the superiority of our instance size adjustment method.

In Table 3, we conduct an ablation study using a different backbone. In the ablation study, we use DarkNet-19 in YOLOv3-tiny. As shown in Table 3, our proposed method improves the detection quality of both YOLOv3-tiny and YOLOv3 networks. This result demonstrates that our proposed method is effective in various networks.

### 4.3. Grad-CAM Result

**Figure 8:**
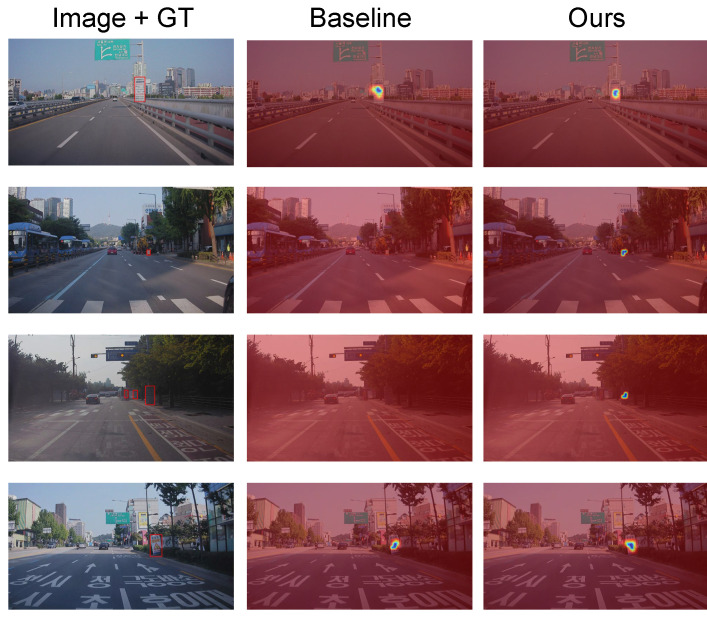
Grad-CAM result. In the first column, input images and corresponding ground truth bonding boxes of the construction sign are shown. In the second and third columns, Grad-CAM results are given. In the Grad-CAM results, high activation values are visualized in blue, while low activation values are visualized in red.

In this subsection, we analyze the effectiveness of our proposed method using Grad-CAM. In Figure 8, we visualize the Grad-CAM [29] results of YOLOv3. To extract Grad-CAM, we compute the gradient of the score for objectness at three different resolution outputs. Then, we average the three activations in each last layer of the decoder. To validate the efficacy of our proposed method, we compare two methods for synthesizing training images. One is to synthesize images simply using naive cut-and-paste method (baseline), and the other one is to synthesize the images using our proposed method. As shown in Figure 8, YOLOv3 trained using a baseline often cannot detect construction signs (second and third rows), while YOLOv3 trained with our proposed method gives accurate activation maps. This result demonstrates that our proposed method helps to learn the discriminative features for construction sign detection.

### 4.4. Effect of Daylight

**Figure 9:**
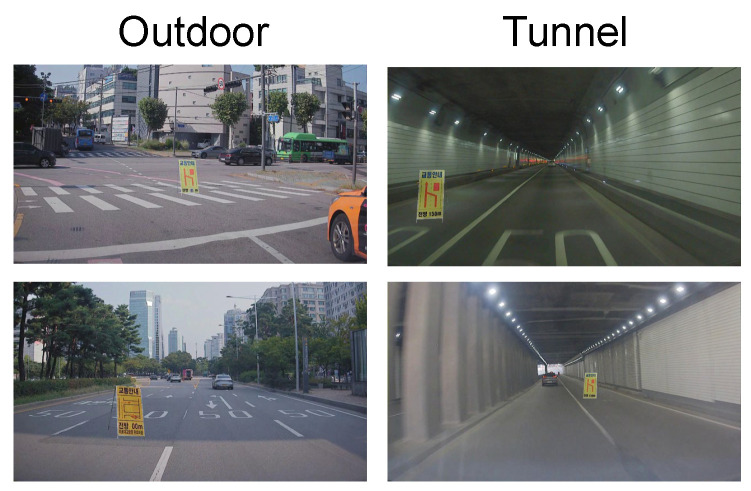
An example of synthesized training set in outdoor and tunnel scenes.

In this subsection, we analyze the effect of daylight and whether on the performance. We build a hierarchical structure in our CSS138 training set by splitting it into two parts: one is captured under sufficient daylight (i.e., outdoor scene), and the other one is captured under low daylight (i.e., tunnel scene). Among 992 road images, 796 images were taken outdoors and 196 images were taken in tunnel. Examples of outdoor and tunnel scenes are given in Figure 9.

With this split, we train detection networks, and the results for the two different daylight conditions are given in Table 4. As shown in the table, daylight significantly contributed to the performance. Specifically, the performance difference between outdoors and the tunnel is about 30%, and the training set captured under sufficient daylight is more effective than the one under low daylight in improving detection performance. Therefore, daylight and weather are crucial for construction sign detection.

### 4.5. Qualitative Analysis

**Figure 10:**
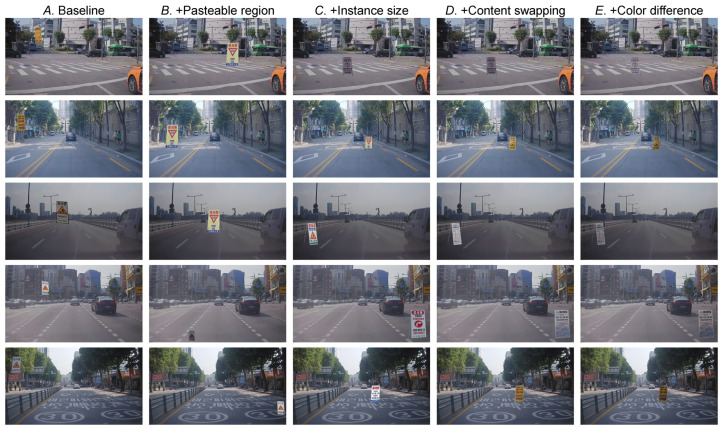
Synthesized training images. For each method, we sampled from the same five background images.

The synthesized training images are shown in Figure 10. Baseline (*A*) often pastes the construction sign on the sky, which never occurs in real-world scenarios. After considering the pasteable region (*B*), the construction sign is placed on the road, but the scale seems very unfamiliar. Our instance size adjustment method (*C*) could address this problem, but the problem of limited sign images remained. Our content swapping (*D*) effectively augments the construction sign images, preventing overfitting. Finally, the color difference (*E*) between the background road image and foreground construction sign image is adjusted to create a realistic image.

**Figure 11:**
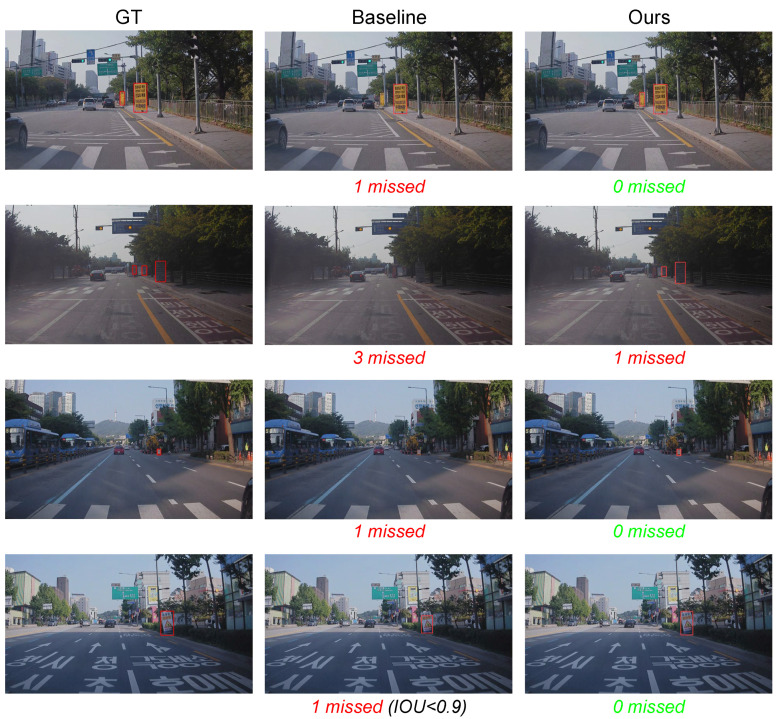
Qualitative results on CSS138 validation set. From left to right, each row shows ground truth (GT), the baseline (naive cut-and-paste), and Ours. For each result on the baseline and ours, we denote the number of missed construction signs.

Figure 11 shows the qualitative results of the proposed methods on the CSS138 validation set, as well as the results of the baseline. As shown in the figure, our method finds small instances (first, second, and third rows) and precisely determines the bounding box of the construction sign (fourth row).

**Figure 12:**
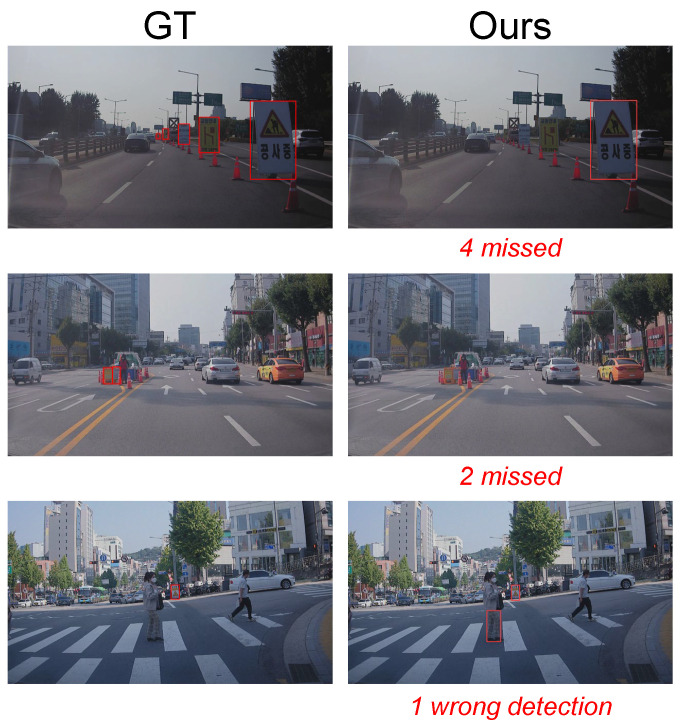
Limitations: if the construction sign is severely occluded, we cannot detect it accurately (first and second rows). A rectangle shape can be detected as a construction sign (third row).

In Figure 12, we present some failure cases, and they show some limitation of our method. The first two rows present false negatives, while the last row present false positive. In the first two rows, construction signs are often missed when they are occluded by other objects such as traffic cones. The last row is the example of the false positive. As can be seen, a rectangular shape object is sometimes detected as a construction sign. We expect that this problem can be solved by various methods, e.g., pre-designing sign shape [10,12], hard example mining [30,31], or learning with strong generalization [32,33].

## 5. Conclusions

In this paper, we have presented a new approach for synthesizing training images for construction sign detection and trained a deep learning detector on them. Since this is the first paper which deals with the construction sign detection, there is not a benchmark set, and we have applied our method to real-world images. Our approach is effective, even when only a few construction sign images are available. Furthermore, our main proposal, content swapping, allows us to use out-domain construction sign data, effectively alleviating the problem of data hunger. To demonstrate the efficacy of our approach, we collected road and construction sign images in person and collected out-domain construction sign images from the Internet. The images used in our experiments are gathered as a dataset CSS138, and we made the dataset available online for the benefit of our community. Even though our method was tested only on the dataset gathered in Seoul, South Korea, we firmly believe that our methods will be applied to other countries and other similar sign-related tasks successfully. Since our content swapping allows us to train networks with a few images, it has the potential to be applied to the few-shot learning field. In this paper, we applied our method only to images, but our proposed method can be extended to videos by applying content swapping and realistic transformations smoothly over time. In addition, our method can be extended to stereo-camera by modeling a construction sign in 3D and projecting it into stereo-view. In addition, a laser scanner sensor can also be considered to measure the distance between the vehicle and the construction sign. The measured distance can improve the quality of the realistic transformations. Furthermore, the future direction of this work would be deciding the action of the autonomous vehicles, after detecting construction signs.

## Figures and Tables

**Table 1:** Experimental results on CSS138 validation set.

Method	AP	AP_50_
*A*. Baseline (cut-and-paste)	60.53	75.84
*B*. + Pasteable region	62.74	77.30
*C*. + Instance size	65.57	82.44
*D*. + Content swapping	68.51	82.73
*E*. + Color difference	70.36	84.98

**Table 2:** Analysis experiment on instance size.

Instance Size	AP	AP_50_
Fixed	62.74	77.30
Random [7]	65.14	80.12
Ours	65.57	82.44

**Table 3:** Ablation study with various backbone networks.

Method	YOLOv3-Tiny (DarkNet-19)		YOLOv3 (DarkNet-53)	
	AP	AP_50_	AP	AP_50_
Baseline	53.40	70.67	60.53	75.84
Proposed	54.95	75.57	70.36	84.98

**Table 4:** Results on two different daylight conditions.

Split	YOLOv3-Tiny (DarkNet-19)		YOLOv3 (DarkNet-53)	
	AP	AP_50_	AP	AP_50_
Outdoor	56.34	76.81	69.32	84.88
Tunnel	16.20	32.29	39.91	55.53

## Data Availability

The raw data supporting the conclusions of this article will be made available by the corresponding author upon reasonable request.
